# p33^ING1b^ methylation in fecal DNA as a molecular screening tool for colorectal cancer and precancerous lesions

**DOI:** 10.3892/ol.2014.1923

**Published:** 2014-02-28

**Authors:** CHUN-GANG HE, QIN-YUAN HUANG, LI-SHENG CHEN, ZHI-AN LING, HONG-GEN WU, HONG-QIANG DENG

**Affiliations:** 1 Department of General and Pediatric Surgery, The People’s Hospital of Guangxi Zhuang Autonomous Region, Nanning, Guangxi Zhuang Autonomous Region 530021, P.R. China; 2 Nursing College of Guangxi Medical University, Nanning, Guangxi Zhuang Autonomous Region 530021, P.R. China; 3 Department of Colorectal and Anal Surgery, The First Affiliated Hospital of Guangxi Medical University, Nanning, Guangxi Zhuang Autonomous Region 530021, P.R. China

**Keywords:** colorectal cancer, precancerous lesions, fecal DNA, p33^ING1b^ methylation, fecal occult blood test

## Abstract

The present study aimed to investigate the feasibility of detecting p33 inhibitor of growth 1b (p33^ING1b^) gene methylation in fecal DNA as a screening method for colorectal carcinoma (CRC) and precancerous lesions. The methylation of p33^ING1b^ was analyzed in fecal samples from 61 patients with CRCs, 27 patients with precancerous lesions (advanced adenoma) and 20 normal individuals by nested methylation-specific polymerase chain reaction (nMSP) and fecal occult blood test. Methylated p33^ING1b^ was detected in 73.77% of CRC patients and 62.96% of adenoma patients. By contrast, only 5% of normal individuals had methylated p33^ING1b^. These results indicated 73.77% sensitivity for detecting CRC, 62.96% sensitivity for detecting precancerous lesions and 95% specificity of the assay for detecting CRCs and precancerous lesions. The detection of p33^ING1b^ methylation status by incubation of DNA contained in agarose beads for bisulfite modification, followed by nMSP, is a promising non-invasive screening method for CRCs and precancerous lesions.

## Introduction

Colorectal carcinoma (CRC) is one of the leading causes of cancer-associated mortality worldwide. Five-year survival is 90% if the disease is diagnosed while still localized (i.e., confined to the wall of the bowel), but only 68% for regional disease (i.e., disease with lymph node involvement) and just 10% if distant metastases are present (1). Detection of early-stage cancer and precancerous lesions is a key measure in reducing the mortality rate. Fecal occult blood tests (FOBT) have reduced the mortality and morbidity of CRC. However, these have a significant false result rate. Alternative screening tests, including flexible sigmoidoscopy and colonoscopy, have been investigated and used as alternatives, but none have been shown to be as cost effective as FOBT in reducing mortality rates. DNA mutation detection is a potentially beneficial test, but has yet to demonstrate superior sensitivity and specificity in a clinical setting (2). Optimized screening methods and markers must be established with high sensitivity and specificity for early-stage cancers and precancerous lesions.

Silencing of tumor suppressor genes by promoter methylation is a common feature of cancer and is recognized to play a crucial role in human tumors. DNA methylation in CRCs has been studied extensively and numerous genes specifically affected by CpG methylation have been identified, including death-associated protein kinase (DAPK) (3), adenomatous polyposis coli, Ras-associated family 2A, Wnt inhibitory factor 1 (4), secreted frizzled-related protein 2 (5), P16, DAPK1, hypermethylated in cancer 1, *O*-6-methylguanine-DNA transferase (6), GATA4, GATA5 (7), thrombospondin 1 (8), A-kinase anchor protein 12 (9) and WNT5A (10). These investigations demonstrated that promoter CpG methylation is a frequent event and often occurs early during CRC carcinogenesis. Aberrantly methylated DNA has also been proposed as a potential tumor marker (11).

Additionally, it has been demonstrated that DNA hypermethylation can be detected in DNA from the feces of CRC patients, suggesting that fecal DNA methylation analysis is a promising approach to non-invasive screening for early colorectal lesions (12–15). Although these reports have confirmed the potential for early detection of colon cancer-derived aberrantly methylated DNA in feces, the majority of these investigations analyzed only a small number of samples. Current detection methods and their sensitivity for known markers are not optimal (16,17). Thus, the development of improved screening methods and identification of new cancer markers is highly desirable.

The p33 inhibitor of growth 1b (p33^ING1b^) gene has been identified as a novel growth inhibitor and tumor suppressor gene, with reduced expression in ovarian, esophageal, gastric, brain and breast cancers. However, mutation of the p33^ING1b^ gene is rare (18–22). The p33^ING1b^ gene promoter region is rich in CpG dinucleotides (23), suggesting that other mechanisms, including DNA methylation, may contribute to the reduced expression of this gene. In the present study, the methylation of p33^ING1b^ in feces from patients with CRCs and precancerous lesions was analyzed and compared with normal controls. In addition, the sensitivity and specificity of this test were compared with those of the immunochemical FOBT (IFOBT), and the feasibility of detecting hypermethylation in feces as a non-invasive screening tool for CRCs and precancerous lesions was evaluated.

## Materials and methods

### Patients and specimens

In total, 61 CRC patients, 27 advanced adenoma (AA) patients and 20 patients with endoscopically normal colons undergoing colonoscopy for routine clinical examination at the First Affiliated Hospital of Guangxi Medical University (Nanning, China) were enrolled in this study. Fecal specimens were collected by patients, sent to the laboratory within 2 h after defecation and stored at −80°C until analyzed. In addition, 37 cancer tissue samples resected by surgery were obtained from the 61 CRC patients. This study was approved by the ethical committee of Guangxi Medical University (Nanning, China). Written informed consent was obtained from the families of the patients.

### IFOBT

All stool samples were examined using a single IFOBT with MagStream HemSp (Fujirebio, Tokyo, Japan), an immunochemical test for human hemoglobin. The FOBT was performed at the laboratory of the First Affiliated Hospital of Guangxi Medical University.

### Fecal DNA isolation

Fecal DNA was isolated by the QIAamp DNA Stool mini kit (Qiagen, Hilden, Germany). The extracted DNA was quantitated by ultraviolet detection and stored at −20°C.

### CRC tissue DNA isolation

Tissue uvDNA isolation was performed by the conventional method of phenol/chloroform treatment followed by proteinase K digestion. The extracted DNA was stored at −20°C.

### Bisulfite modification

The bisulfite modification procedure was performed according to Olek *et al* (24) and Zhang *et al* (25) with minor modifications. In brief, 500 ng genomic DNA was denatured by incubation in 0.3 M NaOH for 15 min at 37°C, then mixed with 2 volumes 2% low melting point agarose. Agarose/DNA mixtures were pipetted into chilled mineral oil to form agarose beads. Each bead was placed in an individual tube to which 200 *μ*l aliquots of 5 M bisulfate solution were added [2.5 M sodium metabisulfite (Sigma, St. Louis, MO, USA) and 100 mM hydroquinone (Sigma); pH 5.0]. The reaction mixture was then incubated in the dark for 16 h at 50°C. Treatment was completed by equilibration against 1 ml Tris*-*EDTA buffer followed by desulfonation in 500 *μ*l NaOH (0.2 M). Finally, the beads were washed with 1 ml H_2_O and then used directly in polymerase chain reaction (PCR).

### Nested methylation-specific PCR (nMSP)

Blood DNA treated *in vitro* with CpG methyltransferase (M.SssI) (Fermentas, Hanover, MD, USA) was used as a positive control for methylated alleles, and placental DNA was used as a negative control for the nMSP assay. The bisulfite-modified DNA underwent nMSP in a blind manner using primer pairs designed to specifically amplify the methylated or unmethylated alleles of p33^ING1b^. The sequences of PCR primers specific for methylated and unmethylated alleles of p33^ING1b^ and the sizes of the expected PCR products are summarized in Table I (22).

Briefly, 3 *μ*l bisulfite-modified DNA was added in a final volume of 25 *μ*l PCR mixture containing 2.5 *μ*l 10X PCR buffer, 2 *μ*l deoxynucleotide triphosphates (2.5 mmol/l), 0.5 *μ*l each nested primer (10 *μ*mol/l) and 1 unit Long *Taq* polymerase (Tiangen Biotech (Beijing) Co., Ltd., Beijing, China). The PCR amplification protocol for stage 1 was as follows: 94°C for 4 min, followed by 35 cycles at 94°C for 30 sec, specific annealing temperature 53°C for 30 sec and 72°C for 30 sec, and finally 72°C for 5 min. For stage 2 amplifications [PCR with the methylation-specific primers (Sangon Biotech (Shanghai) Co. Ltd., Shanghai, China)] 1 *μ*l first-step PCR products was added in a final volume of 25 *μ*l PCR mixture: 2.5 *μ*l 10X PCR buffer, 2 *μ*l deoxynucleotide triphosphates (2.5 mmol/l), 0.5 *μ*l each MSP primer (10 *μ*mol/l) and 1 unit *Taq* polymerase (Takara, Dalian, China). The PCR amplifications were performed at 95°C for 4 min, followed by 35 cycles at 94°C for 30 sec, specific annealing temperature (62.5°C for the methylated-p33^ING1b^ primer and 58°C for the unmethylated-p33^ING1b^ primer) for 30 sec and 72°C for 25 sec, and finally at 72°C for 5 min. nMSP products were analyzed by 2% agarose gel electrophoresis stained with ethidium bromide.

### Statistical analysis

Statistical probabilities were analyzed by 2×2 contingency tables using binomial distribution of differences and Pearson’s χ^2^ test. Calculations were performed with SPSS 13.0 software (SPSS Inc., Chicago, IL, USA). P<0.05 was considered to indicate a statistically significant difference.

## Results

### Detection of p33^ING1b^ methylation in fecal DNA

**Figure 1:**
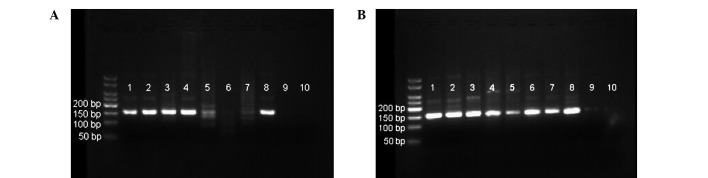
(A) Methylated and (B) unmethylated p33 inhibitor of growth 1b as revealed by nMSP in fecal DNA from patients with CRC. Samples 1–7 were fecal samples from CRC patients and samples 8, 9 and 10 were positive, negative and blank controls for nMSP, respectively. CRC, colorectal cancer; nMSP, nested methylation-specific polymerase chain reaction.

nMSP was performed on all 108 samples, including 37 CRC tissues with matched fecal samples. The correlation of p33^ING1b^ methylation between the DNA from CRC tissues and fecal DNA was examined first (Fig. 1). Of 37 cases, 31 (84%) and 29 (78%) exhibited p33^ING1b^ methylation in the tissue and matched fecal samples, respectively. The coincident rate of p33^ING1b^ methylation status in tissues and the matched feces was 94.6%, with significant correlation (R=0.838; P<0.01; Table II).

An overview of the frequency of p33^ING1b^ methylation in the investigated fecal DNA is provided in Table III. The positive rates of p33^ING1b^ promoter methylation in fecal DNA from 61 sporadic CRC patients and 27 AA patients were 73.77 and 62.96%, respectively. When compared with the control group (5%), there were statistically significant differences (P<0.01) (Table III). The methylation of p33^ING1b^ in the 61 CRC patients was analyzed for association with clinicopathological data. No correlations were identified between the methylation of p33^ING1b^ in feces and gender, age, tumor location, pathological differentiation, lymph node metastasis, distant metastasis and Dukes’ stage (Table IV).

The positive detection rates of FOBT in 61 CRCs, 27 AA cases and 20 control patients were 49.18, 33.33 and 10%, respectively (Table V). The positive rate of FOBT in cases of CRC was significantly higher than in the control group (P<0.01). However, there was no statistically significant difference between FOBT results in cases of AA and the control group (P>0.05).

The sensitivity, specificity, crude accuracy and Youden’s index of the test for p33^ING1b^ methylation in fecal DNA were compared with those of the FOBT (Table VI) (26). Youden’s index was used to measure the effectiveness of the diagnostic marker and is as follows: Youden’s index (J) = Se(c) + Sp(c) - 1 (where Se is senstivity and Sp is specificity). The p33^ING1b^ methylation test had a sensitivity of 73.77% for CRCs and 62.96% for detecting AAs, and a specificity, crude accuracy and Youden’s index of 95%, 84.26% and 0.75, respectively. The FOBT had a sensitivity of 49.18% for CRCs and 33.33% for AAs, and a specificity, crude accuracy and Youden’s index of 90%, 52.78% and 0.44, respectively (Table VI). The sensitivities of the p33^ING1b^ methylation test for CRCs and AAs were higher than those of FOBT (P<0.01 and P<0.05, respectively). There was no statistically significant difference between the specificities of the two methods (P>0.05). The crude accuracy of testing for p33^ING1b^ methylation in fecal DNA was higher than that of FOBT (P<0.01).

## Discussion

Since misregulation of gene expression by aberrant DNA methylation is a well-characterized event in tumor biology and has been extensively documented for CRC (3–10), identification of aberrantly methylated genes is a promising strategy for research, diagnostics and therapeutics (27). However, specific disadvantages associated with fecal DNA testing render it unsuitable for use in general population screening for CRC at present. Further study is required to optimize the methods and the selection of epigenetic markers. In addition, simplified, inexpensive and automatized assays may be important for population-based screening (28).

In the present study, the feasibility of detecting methylated fecal DNA by nMSP as a screening tool for CRCs and precancerous lesions was explored. Although the routine method of DNA bisulfite modification with MSP is the most common method for detecting DNA methylation in previous studies (14,29,30), this method has not been optimized: The process is complicated, has low sensitivity (detects 1 methylated allele in up to 1,000 unmethylated alleles) due to sample damage and loss during bisfulfite modification, and is expensive when a commercial kit is used. Grunau *et al* (31) demonstrated that for the majority of routine PCR, 50 ng human template DNA yields sufficient PCR product. The authors found that ~90% of the template DNA is lost during treatment with bisulfite. In the present study, the concentration of fecal DNA extracted by the commercial kit was 67.35±13.17 ng/l, while some samples did not satisfy the requirements for bisulfite treatment and MSP. Under these circumstances, this method may result in an abnormally low detection rate of fecal DNA methylation. As demonstrated by Olek *et al* (24), bisulfite modification by incubation of DNA contained in agarose beads, followed by nMSP to detect DNA promoter methylation, is easy to perform and high levels of sensitivity can be reached. Treatment of DNA contained in agarose beads minimizes DNA degredation and prevents incomplete conversion of the DNA. The authors also succeeded in amplifying fragments of up to 3 kb, which cannot be achieved using conventional methods. The improved MSP procedure incorporates a nested, two-stage PCR approach, which is more sensitive than the conventional method (detects 1 methylated allele in up to 50,000 unmethylated alleles) (32). In the current study, as little as 10 pg DNA was amplified by treatment within agarose beads followed by nMSP, and even by general Taq polymerase can succeeded in nMSP. This level of sensitivity allows determination of methylation patterns in small fecal samples, advantageous for a high-sensitivity screening method for CRCs. In addition, this study demonstrated that DNA extracted from feces is of sufficient quality and quantity for the detection of DNA methylation by this method. This is also a less expensive test compared with commercial kits for bisulfite modification and PCR.

Next, the methylation status of p33^ING1b^ was investigated in the fecal DNA of CRC patients and the matched tissues and precancerous lesions. To examine the correlation of methylation status between fecal DNA and DNA from cancerous tissue, 37 matched pairs of samples were tested for methylation. The nMSP results were consistent between the paired fecal and tissue samples (R=0.838; P<0.01) indicating that the test for p33^ING1b^ methylation in fecal DNA may reflect the true methylation status of CRCs.

This study revealed that the p33^ING1b^ methylation test, assessed in independent sets of patients, has sensitivities of 73.77 and 62.96% for identifying patients with CRC and precancerous lesions, respectively, while only 5% of control group samples tested positive for p33^ING1b^ methylation. No correlation was identified between p33^ING1b^ methylation status in fecal DNA and any clinical or pathological characteristics of cancer, indicating that this test is as sensitive in early-stage CRCs as in late-stage CRCs. Previous studies (14,30) have demonstrated that aberrant gene methylation may occur in the early stages of CRCs and even in precancerous lesions, and that this can be detected in feces, which has potential value in the noninvasive and early diagnosis of colorectal neoplasms. However, the low sensitivity and specificity of the assays suggest the need to develop more powerful techniques. The present results demonstrate that methylation of the p33^ING1b^ gene in feces can be considered as a biomarker for CRCs and precancerous lesions, however, these findings must be confirmed or improved upon in studies with larger sample sizes.

FOBT is the most widely used method to screen for CRCs. Although FOBT is valuable as a noninvasive screening method that reduces the risk of CRC-associated mortality (33,34), it has limited sensitivity that leads to numerous CRCs remaining undetected (35–37). This limitation shows that FOBT is not the best method for screening CRCs. By contrast, using aberrant gene methylation as a molecular marker offers a potentially powerful approach to population-based screening for CRCs and precancerous lesions (28). In the present study, the efficacies of the fecal p33^ING1b^ methylation test and FOBT for the identification of CRCs and precancerous lesions were compared. FOBT had sensitivities of 49.18, 33.33 and 10% for CRCs, precancerous lesions and the normal control group, respectively, which were lower than those for the detection of p33^ING1b^ methylation in feces (P<0.05 for AA and P<0.01 for CRCs). However, the two methods had high specificity for CRCs and precancerous lesions (≥90%) and the sensitivity of FOBT for CRCs was greater than that for the control group (P<0.01). No statistically significant difference in FOBT sensitivity for precancerous lesions and the normal control group was identified (P>0.05). These results suggest that FOBT may be a useful method for screening CRCs but not for precancerous lesions, and that the detection of p33^ING1b^ methylation in feces may be more effective than FOBT in screening for CRCs and precancerous lesions. It is clear from this study that testing for p33^ING1b^ methylation in fecal DNA exhibited a higher sensitivity than FOBT without reduced specificity.

In conclusion, the results of the present study demonstrate the value of bisulfite modification of DNA contained in agarose beads, followed by nMSP, to detect fecal DNA promoter methylation, as a noninvasive and simple test, and suggest p33^ING1b^ methylation as a potential biomarker in screening for CRCs and precancerous lesions.

## Figures and Tables

**Table I:** Primers for nested methylation-specific polymerase chain reaction.

Primer names	Sequences, 5′-3′	Size, bp
Nested primer		44
Forward	AGATAAGGTTTAGGGAAGGYGTT	
Reverse	AACAACCRCAATAACCAATCTACT	
Methylated p33^ING1b^ primer		151
Forward	CGGATGGCGTAGGCGCGGGAGTC	
Reverse	CCGAACACGAACGAAAATAACGACGC	
Unmethylated p33^ING1b^ primer		151
Forward	TGGATGGTGTAGGTGTGGGAGTT	
Reverse	CCAAACACAAACAAAAATAACAACACA	

p33^ING1b^, p33 inhibitor of growth 1b.

**Table II:** Consistency analysis of p33 inhibitor of growth 1b methylation in tissues and matched fecal samples of colorectal cancer.

	Cancerous tissues		
		
Fecal DNA samples	Methylated	Unmethylated	Total
Methylated	29	0	29
Unmethylated	2	6	8
Total	31	6	37

Spearman’s correlation, R=0.838; P<0.01 vs. no correlation.

**Table III:** p33^ING1b^ promoter methylation in fecal DNA of CRC and AA patients and the control group.

		p33^ING1b^ methylation, n (%)	
		
Fecal DNA	Samples, n	Methylated	Unmethylated
CRC^a^	61	45 (73.77)	16 (26.23)
AA^a^	27	17 (62.96)	10 (37.04)
Control	20	1 (5.00)	19 (95.00)

a P<0.01, vs. control group.

AA, advanced adenoma; CRC, colorectal cancer; p33^ING1B^, p33 inhibitor of growth 1b.

**Table IV:** Correlations between clinical parameters and the methylation of p33^ING1b^ promoter in fecal DNA from colorectal cancer patients.

		p33^ING1b^ methylation, n (%)			
				
Group	Patients, n	Methylated	Unmethylated	χ^2^	P-value
Gender				0.176	0.674
Male	37	28 (75.68)	9 (24.32)		
Female	24	17 (70.83)	7 (29.17)		
Age, years				0.434	0.510
≤58	30	21 (70.00)	9 (30.00)		
>58	31	24 (77.42)	7 (22.58)		
Tumor location				79.31	0.349
Colon	29	23 (79.31)	6 (20.69)		
Rectum	32	22 (68.75)	10 (31.25)		
Pathological differentiation				1.490	0.222
High or middle	38	26 (68.42)	12 (31.58)		
Low	23	19 (82.61)	14 (17.39)		
Lymph node metastasis				0.218	0.640
Presence	22	17 (77.27)	5 (22.73)		
Absence	39	28 (71.79)	11 (28.21)		
Distant metastasis				0.085	0.771
Presence	9	9 (81.82)	2 (18.18)		
Absence	52	36 (72.00)	14 (28.00)		
Dukes’ stage				0.617	0.432
A/B	33	23 (69.70)	10 (30.30)		
C/D	28	22 (78.57)	6 (21.43)		

p33^ING1b^, p33 inhibitor of growth 1b.

**Table V:** FOBT detection rates in CRC, AA and the control group.

		FOBT, n (%)	
		
Group	Samples, n	Positive	Negative
CRC^a^	61	30 (49.18)	31 (50.82)
AA	27	9 (33.33)	18 (66.67)
Control group	20	2 (10.00)	18 (90.00)

a P<0.01, vs. control group.

AA, advanced adenoma; CRC, colorectal cancer; FOBT, fecal occult blood test.

**Table VI:** Comparisons of p33 inhibitor of growth 1b methylation testing of fecal DNA for CRCs and AAs with FOBT.

			CRC and AA		
			
Group	CRC sensitivity, %	AA sensitivity, %	Specificity, %	Crude accuracy, %	γ
Fecal DNA detection	73.77	62.96	95	75.00	0.70
FOBT	49.18	33.33	90	52.78	0.44
χ^2^	7.787	4.747	0.360	11.559	
P-value	0.005	0.029	0.548	0.001	

AA, advanced adenoma; CRC, colorectal cancers; FOBT, fecal occult blood test.
